# Individualization of *Pinus radiata* Canopy from 3D UAV Dense Point Clouds Using Color Vegetation Indices

**DOI:** 10.3390/s22041331

**Published:** 2022-02-09

**Authors:** Antonio M. Cabrera-Ariza, Miguel A. Lara-Gómez, Rómulo E. Santelices-Moya, Jose-Emilio Meroño de Larriva, Francisco-Javier Mesas-Carrascosa

**Affiliations:** 1 Centro de Investigación y Estudios Avanzados del Maule, Universidad Católica del Maule, Avenida San Miguel 3605, Talca 3460000, Chile; 2 Centro de Desarrollo del Secano Interior, Facultad de Ciencias Agrarias y Forestales, Universidad Católica del Maule, Avenida San Miguel 3605, Talca 3460000, Chile; rsanteli@ucm.cl; 3 Centro de Investigaciones Aplicadas al Desarrollo Agroforestal S.L., 14001 Córdoba, Spain; mlara@idaf.es; 4 Department of Graphic Engineering and Geomatics, Campus de Rabanales, University of Cordoba, 14071 Córdoba, Spain; ir1melaj@uco.es (J.-E.M.d.L.); ig2mecaf@uco.es (F.-J.M.-C.)

**Keywords:** unmanned aerial vehicle, progressive triangulated irregular network, color vegetation index

## Abstract

The location of trees and the individualization of their canopies are important parameters to estimate diameter, height, and biomass, among other variables. The very high spatial resolution of UAV imagery supports these processes. A dense 3D point cloud is generated from RGB UAV images, which is used to obtain a digital elevation model (DEM). From this DEM, a canopy height model (CHM) is derived for individual tree identification. Although the results are satisfactory, the quality of this detection is reduced if the working area has a high density of vegetation. The objective of this study was to evaluate the use of color vegetation indices (CVI) in canopy individualization processes of *Pinus radiata*. UAV flights were carried out, and a 3D dense point cloud and an orthomosaic were obtained. Then, a CVI was applied to 3D point cloud to differentiate between vegetation and nonvegetation classes to obtain a DEM and a CHM. Subsequently, an automatic crown identification procedure was applied to the CHM. The results were evaluated by contrasting them with results of manual individual tree identification on the UAV orthomosaic and those obtained by applying a progressive triangulated irregular network to the 3D point cloud. The results obtained indicate that the color information of 3D point clouds is an alternative to support individualizing trees under conditions of high-density vegetation.

## 1. Introduction

Traditional forest inventory systems rely primarily on field data and statistical estimators based on sample design. These methods can provide estimates of inventory variables, although they come at a significant economic cost [1]. In addition, field-scale data collection is time-consuming and offers uncertain results due to the variability of tree canopies in forests or plantations and the difficulty of adapting to geometric patterns such as cones or ovoids to be able to map them in geographic information systems [2]. In addition, data collected from field measurements are often associated with sampling and observation errors [3].

In recent years, remote sensing has become an increasingly reliable discipline in geomatic techniques to determine parameters of interest in forests, both at mass and individual tree levels [4]. The images used can be registered by sensors on-board three types of platforms: satellite, manned, and unmanned air platforms. Firstly, earth observation (EO) programs have been used in natural resource management to obtain images of medium [5] or high spatial resolution [6], offering data with different spatial, spectral, radiometric, and temporal resolution based on different technologies [7]. Furthermore, its global coverage reduces the intensity of sampling, and thus economic and temporary costs, and provides data on inaccessible or difficult-to-access areas. However, satellite platforms have some drawbacks. Passive sensors are dependent on meteorological conditions, and there are limitations on acquiring traditional set of forest parameters obtained by the classical method, such as canopy diameter or basal areas. Nevertheless, these images have been widely used in forestry activities [5,6,8]. Manned aerial platforms allow forest inventory to be carried out on much larger areas compared to what is achievable with traditional field methods [9]. This includes the use of passive sensors, such as RGB [10], multispectral [11], hyperspectral [12], and thermographic [13] sensors, as well as active sensors, such as light detection and ranging (LiDAR) [14], which has become a tool for forest inventory in many countries around the world [15,16,17,18]. However, the high economical cost of manned aerial platforms makes it difficult to carry out continuous monitoring of an area of interest [19]. Unmanned aerial vehicles (UAV) are increasingly being used in forestry [20,21,22,23,24,25]. These platforms allow acquisition of data with very high spatial and temporal resolution, which can be used for mapping forest areas [25] to identify species or degree of stress and/or diseases [23,26] as well as individual tree identification by means of RGB [27], hyperspectral [28], multispectral [29], or LiDAR [30,31] sensors. Therefore, UAVs present a good alternative that can be used to register remote RGB, multispectral, hyperspectral, and thermographic images at the right moment and in a repeated manner [32,33].

Forest inventory remains a challenge, with the detection and delineation of individual tree crowns (ITCs) being a prerequisite to estimate parameters such as diameter, height, and biomass, among other variables [34,35]. Different ITC methods, such as passive [36], active [37], and multiple data sources [38], have been developed. Tree location algorithms include template matching, imagen binarization, and local maximum filtering techniques, among others [39]. On the other hand, delineate tree crown algorithms can be categorized into valley following, region growing, and watershed segmentation [39]. In this context, data for ITC can be obtained from either passive sensors [40] or LiDAR [41]. Passive sensors and photogrammetry techniques allow forest inventory metrics to be determined because of their ability to provide orthomosaics and 3D point clouds, which are produced from stereoscopic images based on structure from motion (SfM) [42]. However, unlike LiDAR point clouds, they can only produce accurate digital surface model (DSM) in dense forest because of their inability to penetrate the foliage to reach the ground [43]. Therefore, an external digital elevation model (DEM) is needed to produce a canopy height model (CHM).

The information derived from 3D dense point clouds, whether from active or passive sensors, starts from the correct individualization of trees. For this, the first step is to classify ground points. The classification quality of 3D dense point clouds generated using images from passive sensor on-board UAVs in dense forest areas is poor, offering unsatisfactory results and significantly affecting other processes [14]. Therefore, accurate DEM generation is a prerequisite for accurate characterization of forest information using photogrammetric 3D dense point clouds. The results obtained can be comparable even to those acquired with LiDAR data [10,44].

A common strategy for tree individualization is to convert 3D dense point clouds, mainly derived from LiDAR flights, into a CHM or another tree height model and then find local minimum height values [45,46,47,48]. In this case, depending on the sensor type, the difficulty lies in the need to classify points belonging to the ground, which will allow the point cloud to be processed correctly for the individualization of trees [49,50]. Once the point clouds have been processed and filtered, there are various algorithms for the detection and segmentation of trees, such as the local maximum algorithm [30], template matching [51], watershed segmentation [3], region growing [52], and crown delineation based on optimized object recognition, treetop identification, and hill-climbing (COTH) algorithm [53], among others. Methods for the individualization of canopies using passive sensors and photogrammetry techniques can be distinguished into two groups: those based on using data shapes derived from point clouds [11] and those using orthomosaics. In this context, Sperlich et al. [54] developed point clouds from aerial imageries based on UAVs and achieved an individualization precision of 87.68% using a watershed algorithm in a dense coniferous forest. Kattenborn et al. [55] updated the algorithm of Sperlich et al. [54], geometrically classifying UAV-derived point clouds and identifying densely scattered palm trees in a 9.4 ha study area with abundant undergrowth and other trees with an overall accuracy of 86.1%. All the methodologies outlined above are based on metric parameters, such as slope, minimum distance, or height. Using a different approach, Mesas-Carrascosa et al. [2] applied color vegetation indices on 3D dense points clouds to determine the height of a plant species, vines in this case, automatically detecting and classifying points belonging to the vegetation class to later determine the height of vines with reference to heights of the points classified as ground.

The objective of this study was to evaluate the use of a color vegetation index in *Pinus radiata* canopy individualization processes using CHMs obtained from high-density 3D point clouds generated by RGB sensors on-board UAVs.

## 2. Materials and Methods

### 2.1. Study Area

**Figure 1:**
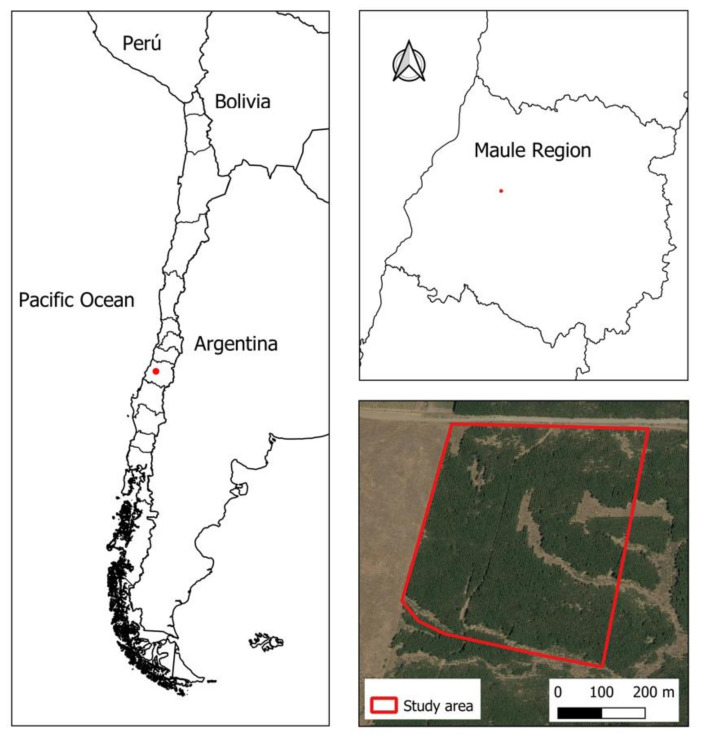
Study area.

The present research was performed on a 1998 *Pinus radiata* D Don plantation (35°28′20.32″ S, 71°48′55.41″ W, WGS84) covering an area equal to 23.7 hectares, located in the Querquel area (Talca, Chile) (Figure 1) at a height of 93 m above sea level. The mean annual temperature is equal to 14.2 °C, and the mean annual rainfall is 845 mm. The plantation is located on soils from the Pocillas Association series, characterized by having a moderately fine texture and being deep (more than 100 cm), gently rolling, slightly stony without erosion, moderately acidic (pH between 5.6 and 6), nonsaline, and nonalkaline [56].

**Figure 2:**
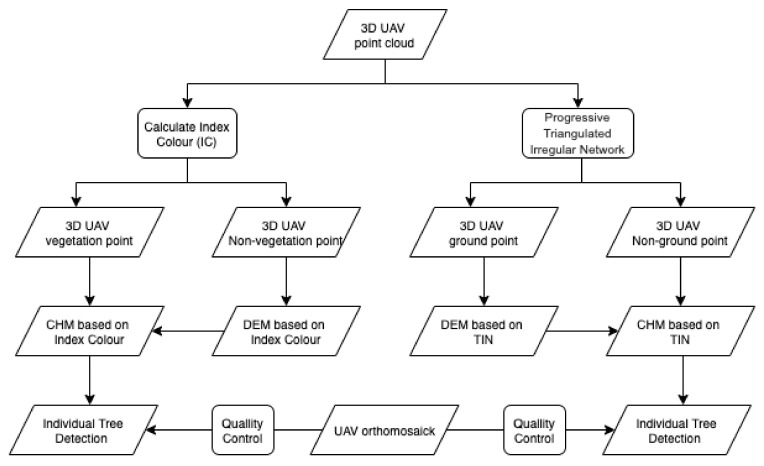
Workflow.

Figure 2 shows the workflow followed in the present study. Once the UAV flights were performed, we proceeded to process them to obtain a 3D dense point cloud and an orthomosaic. A color vegetation index (CVI) was applied to the point cloud to differentiate the points belonging to vegetation from nonvegetation classes. The latter were used to create a DEM that will hereafter be referred to as DEM based on CVI (DEM-CVI). On the other hand, ground points from original 3D dense point cloud were classified by a progressive triangulated irregular network (TIN) algorithm, and a DEM was generated (DEM-TIN). From each DEM, a CHM was derived and an automatic canopy identification procedure was applied. Finally, the results were evaluated by contrasting with the canopies manually identified in the orthomosaic generated from the UAV flight.

### 2.2. UAV Flights

The images were acquired on 31 March 2020 using a DJI Plahtom4 advanced platform (SZ DJI Technology Co., Shenzhen, China). The on-board sensor for acquiring images was an RGB sensor (R: red; G: green; B: blue) with a sensor size of 1/2.3” CMOS, a field of view lens equal to 94° lens, and a focal length of 20 mm, allowing images with an image size of 4000 × 3000 pixels to be registered. The flight height was 100 m above ground level. A crossover UAV flight was planned with flightlines in N–S and E–O directions. The images were registered in continuous mode to 2 s intervals and a speed of 4.5 m ×s^−1^, resulting in a side and forward lap equal to 95% and 70%, respectively. The selection of these overlapping percentages between images allowed an adequate 3D reconstruction of the study area [57].

Five ground control points (GCPs) were placed, one in each corner and the other in the center of the study area. Then, aerotriangulation was calculated, allowing accurate and precise determination of the absolute orientation, position, and orientation of each image of the photogrammetric block. Subsequently, the 3D dense point cloud was generated using structure from motion (SfM) techniques. This methodology has been validated in previous research projects [58]. In addition, an orthomosaic was generated. We used Pix4Dmapper software (Pix4D S.A., Prilly, Suiza) for photogrammetric processing.

### 2.3. Ground Points Classification and Digital Elevation Model

Two different strategies were applied in point classification based on (a) color vegetation index and (b) point elevation. In the generation of the 3D dense point cloud, each of the points was associated with an RGB color value resulting from projecting these onto the stereoscopic model where applicable. Based on these RGB values, a classification was performed to discriminate between points belonging to the vegetation class and nonvegetation class. The nonvegetation class collected points that belong to the ground as well as shadows and other artificial elements. Based on our previous research experience [2], the normal green-red difference index (NGRDI) [59] using Equation (1) was calculated for each point based on RGB values.

(1) $$NGRDI=\left(g-r\right)/\left(g+r\right)$$

Thus, taking into account the information of each point referred to the RGB color space and before calculating the index, a standardized color space was performed [60]. As a result, the normalized color components r, g, and b were found in the range [0, 1] as calculated using Equations (2)–(4):

(2) $$r=R/\left(R+G+B\right)$$

(3) $$g=G/\left(R+G+B\right)$$

(4) $$b=B/\left(R+G+B\right)$$

where R, G and B are the normalized RGB values in the range [0, 1] obtained using Equations (5)–(7):

(5) R=R/R_max 

(6) G=G/G_max

(7) B=B/B_max

where, R_max, G_max, and B_max are all equal to 255 for images with 24 radiometric bit resolution.

Through a script developed in MATLAB, the original 3D RGB point cloud was converted into a grayscale, with the value of the NGRDI index being the value of the attribute for each point. The distribution of NGRDI values of the points followed a binomial distribution representing the vegetation and nonvegetation classes. The next step was to analytically determine the value of the separation threshold between both classes using the Otsu method [61]. This method consists of analyzing the histogram of the NGRDI values to search for the separation of the two normal distributions present in the bimodal distribution. As a result, two 3D point clouds were obtained from the original, one representing points belonging to the vegetation class and the other to the nonvegetation class.

On the other hand, based on point elevation, ground points were classified using a progressive triangulated irregular network (TIN) densification algorithm using LAStools [62]. Although there are different filtering algorithms that offer good results [63], the progressive TIN algorithm is suitable for working with 3D UAV point clouds [64] as it is robust against the random noise of these point clouds [65]. According to Mohan et al. (2017) [66], the parameters settings were as follows: step 10 m, bulge 0.5, spike 1 m, and offset 0.05 m.

As a result, two DEMs with a spatial resolution equal to 1 m were generated from both classifications. Based on RGB values, points classified as nonvegetation were used to obtain a DEM-CVI, while points classified as ground were used to generate a DEM-TIN.

### 2.4. Canopy Height Model and Individualization of Canopies

From the two previously generated DEMs, two CHMs (CHM-CVI and CHM-TIN) were determined. Each CHM was created by assigning the highest elevation point within 1 m to the center of the grid cell in each grid, which were processed using the rLiDAR package. First, CHM was filtered by 3 × 3 pixel window Gaussian filter to search for apices [46,66]. Subsequently, the height from which the processing interrupts the search for new trees was established at 7 m after verifying with greater heights, which obtained worse results. A maximum canopy radius of 2.5 m was also established according to what was observed on the field. The exclusion parameter, which takes values between 0 and 1, represents the percentages of excluded pixels. A value of 0.5 will exclude all the pixels of a single tree that has a height of less than 50% of the maximum height of the same tree. After several tests, this value was set to 0.66. Finally, the projected area on the ground of the individual tree canopies detected from the CHM was delineated, and the coordinates of the centroids of the individualized canopy areas were calculated. For the individualization of canopies, FUSION [67] and the rLiDAR package were used [68].

To validate the results, 30 random sampled plots were established in the study area. The plots, which were circular shape with a radius of 12.7 m, covered an area of 507 square meters. A visual inspection on the orthomosaic was performed on these plots to identify each of the trees as ground truth to carry out a quality control of the results obtained in the automatic identification processes using both CHMs. In particular, the precision was evaluated in terms of true positive (TP, correct detection), false negative (FN, omission error), and false positive (FP, commission error) as well as with respect to sensitivity (S), precision (P), and F-score (F) as explained in Mohan et al. (2017) [66] using Equations (8)–(10):

(8) $$S=\mathrm{TP}/\left(\mathrm{TP}+\mathrm{FN}\right)$$

(9) $$P=\mathrm{TP}/\left(\mathrm{TP}+\mathrm{FP}\right)$$

(10) $$F=2\times S\times P/\left(S+P\right)$$

In this case, sensitivity is understood as a measure of correctly detected trees as it is inversely related to omission error, precision is the measure of correctly detected trees as it is inversely related to the commission error, and F-score represents the harmonic mean of sensitivity and precision.

## 3. Results

### 3.1. Digital Elevation and Canopy Height Models

**Figure 3:**
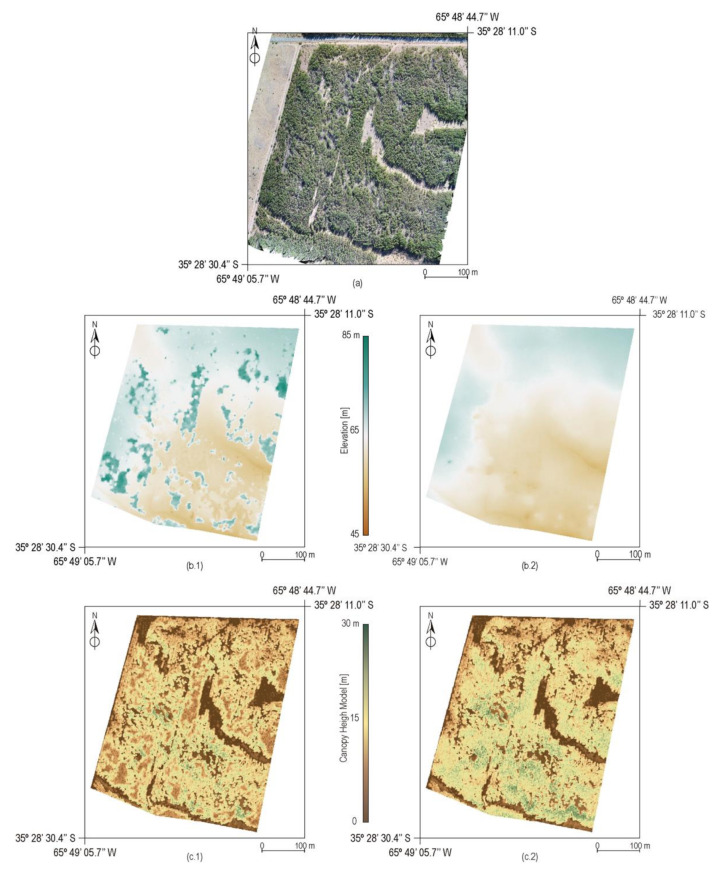
Results of processing: (**a**) orthomosaic of the study area, (**b**) digital elevation model, and (**c**) canopy height Models generated through (1) progressive triangulated irregular network and (2) color vegetation index.

Figure 3 shows the orthomosaic of the study area as well as the DEMs and CHMs generated by the CVI and TIN methods. In addition, Table 1 shows statistics for each digital model. In orthomosaic processing (Figure 3a), about 99 million 3D points were generated, that is, about 78.75 points per m^3^. The spatial resolution of the orthomosaic was 2.8 cm per pixel. As shown, there were areas with dense vegetation where the ground was not visible and areas with less dense vegetation.

In relation to the DEM, in DEM-TIN (Figure 3b.1), there were islands distributed throughout the study area where the elevation rose abruptly. These areas coincided with the presence of dense vegetation. On the other hand, in DEM-CVI (Figure 3b.2), these areas did not appear. Both DEM had different percentiles for the elevation variable, with DEM-TIN having higher percentiles, except for the 50th percentile. These differences increased with increasing percentile. Such differences can be justified because points belonging to the vegetation class were classified as ground in DEM-TIN, thus increasing the value of the terrain elevation represented in the DEM.

**Figure 4:**
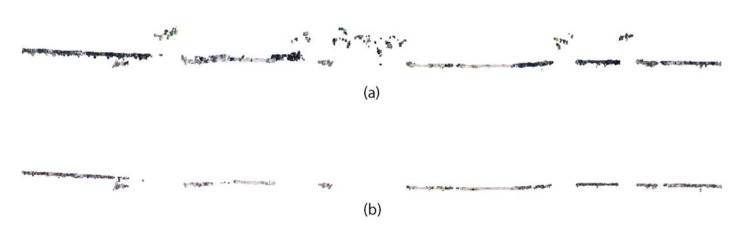
Classification of ground points through (**a**) progressive triangulated irregular network and (**b**) color vegetation index.

On the other hand, both CHMs also showed differences according to the DEM used. The areas that showed a high value of height with respect to the surrounding areas in DEM-TIN presented low values in the case of CHM-TIN (Figure 3c.1). Furthermore, the values of normalized heights were higher for CHM-CVI (Figure 3c.2) than for CHM-TIN. As an example, Figure 4 shows a profile of the points classified as ground taking into account the use of a progressive TIN (Figure 4a) and CVI (Figure 4b). Using progressive TIN (Figure 4a), a group of points that belonged to the vegetation class were classified as ground points and therefore altered the derived DEM and CHM. On the other hand, these points did not appear with CVI (Figure 4b), and DEM and CHM are therefore properly generated.

### 3.2. Individualization of Canopies

**Figure 5:**
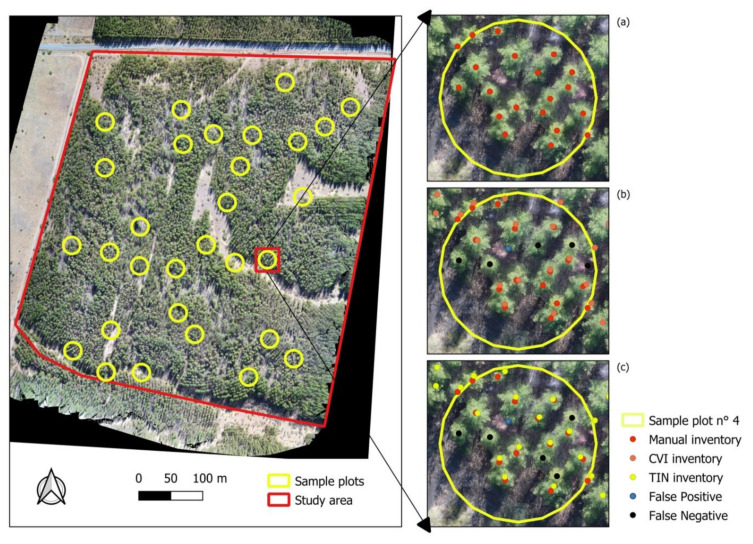
Sample plots in the study area. Detail of plot N° 4: Identification of trees visually (**a**) by color index (**b**) and by original cloud (**c**). Detail of false positives and false negatives.

Figure 5 shows the location of the sample plots in the study area and details of visual individualization in orthomosaic processing (Figure 5a) as well as the results, including false positives and false negatives, of automatic individualization based on CVI (Figure 5b) and TIN (Figure 5c). Table 2 shows the results of the quality assessment for each plot.

A total of 660 individual trees were manually identified in the 30 plots, with an average value of 22 trees per plot. Regarding the number of TPs, a total of 481 trees (72.9%) were correctly detected using CVI compared to 392 (59.4%) using TIN. The number of FP was equal to 8 (1.2%) and 23 (3.5%) for CVI and TIN, respectively. Moreover, the number of FN was lower in the CVI-based classification (171, 25.9%) than in TIN (245, 37.15%). Thus, the average precision obtained for classification by CVI reached a value equal to 0.98 compared to 0.62 obtained by TIN. Similarly, the mean sensitivity and F1-score using CVI was equal to 0.74 and 0.84, respectively, versus 0.62 and 0.74, respectively, using the TIN classification.

Based on these results, better results for sensitivity, accuracy, and F-score were achieved for the classification of 3D point cloud using CVI compared to those obtained by TIN. This indicates that the method of filtering 3D UAV point cloud using CVI in a scenario with high vegetation density provides more accurate results in individual tree identification.

## 4. Discussion

In recent years, several studies have highlighted the potential of remote sensing in forestry. In particular, sensors on-board UAV platforms are an adequate tool in determining the number of trees, height, or biomass [27,57,69,70]. In this paper, we present the utility of CVI in classifying 3D point clouds in vegetation and nonvegetation classes in forestry areas with high density vegetation as a preliminary step to generate a DEM and CHM. The use of CVI has been successfully employed to mainly identify vegetation in images [71], with a few prior cases of it being applied to 3D point clouds [2] and never in forest scenery.

Previous studies have reported an accuracy higher than 80.0% for individual tree detection [72,73,74]. However, these studied forests had low density or flat ground plantations. In particular, our results in canopy mountains were similar to those reported by Guerra-Hernández et al. [10] and much better than those reported by other authors [75] with an accuracy of 67%. On the other hand, recent studies have demonstrated that deep learning methods are an alternative to detect individual trees [76,77]. To our knowledge, CVIs such as NGRDI have not previously been used to automatically classify 3D cloud points in forestry area for individual tree detection. The use of CVIs to classify 3D cloud points to perform DEM and CHM allows a fully automatic method without the need for any manual selection parameter. Therefore, the results depend only on the radiometric information of each of the individual points without any geometric requirement. However, the conditions under which the UAV flight is performed can affect the quality of the results. In addition, the time of day when the UAV flight takes place is important and should preferably be at noon sunlight. Thus, images must be captured under stable weather, light, and shadow conditions. Radiometric quality of 3D point colors, such as color contrast and image contrast [78], can be reduced on cloudy days because of lack of direct sunlight [79]. On the other hand, direct lighting increases contrast and also leads to an increase in the amount of shadows, as does flying on sunny days in the morning and afternoon with low solar angles, which will affect point cloud quality [79].

Modern forestry primarily requires digital forest information, and UAV-based remote sensing offers a promising future in this regard [80]. In addition, the ease of data collection, images with very high spatial and temporal resolution, and low operating costs support data collection with UAV. Future projects should develop tree detection algorithms based on the characteristics of 3D point clouds to include species identification and evaluation of estimation of other characteristics at the tree level, such as DBH and canopy area, which are important and necessary factors to estimate biomass.

## 5. Conclusions

In this work, a new methodology is presented for the individualization of *Pinus radiata* based on the color information of the 3D point clouds generated by RGB sensor images on-board UAVs. The results were compared with those obtained for individualization of trees using progressive triangulated irregular network and with visual tree identification on an orthomosaic. The results obtained indicate that the color information of 3D point clouds is an alternative for the individualization of trees under the conditions of this investigation.

The proposed methodology reveals the potential of cloud-based UAV photogrammetric points for the individualization of trees and forest monitoring. Future research should focus on estimating individual tree attributes, such as canopy height, size, and diameter, and on developing models predictive of estimating aerial biomass and stem volume from UAV images.

## Figures and Tables

**Table 1:** Distribution of percentile heights in digital elevation models (DEM) and canopy height models (CHM) considering the classification of ground points with progressive triangulated irregular network (TIN) and color vegetation index (CVI).

	Height Percentile [m]										
Digital Model	0	10	20	30	40	50	60	70	80	90	100
DEM-TIN	53.30	59.17	60.55	62.05	63.80	64.93	66.20	67.50	68.59	70.98	83.40
DEM-CVI	53.15	58.36	59.47	60.46	62.11	65.66	64.65	65.83	67.10	68.16	74.75
CHM-TIN	0	0.59	4.31	8.59	11.52	13.62	15.27	16.70	18.16	19.91	28.54
CHM-CVI	0	0.75	4.95	11.58	14.34	16.09	17.48	18.72	19.99	21.55	29.01

**Table 2:** The accuracy evaluation for the individualization of trees from the point cloud filtered with color index and progressive triangulated irregular network. TP: true positive; FP: false positive; FN: false negative; S: sensitivity; P: precision; F: F-score.

Plot	Manual Inventory	Color Vegetation Index						Triangulated Irregular Network					
		TP	FP	FN	S	P	F	TP	FP	FN	S	P	F
1	16	16	0	0	1.00	1.00	1.00	11	0	5	0.69	1.00	0.81
2	21	16	0	5	0.76	1.00	0.86	10	1	10	0.50	0.91	0.65
3	9	5	0	4	0.56	1.00	0.71	4	1	4	0.50	0.80	0.62
4	22	11	2	9	0.55	0.85	0.67	6	2	14	0.30	0.75	0.43
5	20	15	1	4	0.79	0.94	0.86	14	1	5	0.74	0.93	0.82
6	28	22	0	6	0.79	1.00	0.88	19	2	7	0.73	0.90	0.81
7	30	25	0	5	0.83	1.00	0.91	16	0	14	0.53	1.00	0.70
8	17	11	1	5	0.69	0.92	0.79	11	1	5	0.69	0.92	0.79
9	26	16	0	10	0.62	1.00	0.76	13	2	11	0.54	0.87	0.67
10	25	18	0	7	0.72	1.00	0.84	18	0	7	0.72	1.00	0.84
11	25	13	0	12	0.52	1.00	0.68	11	0	14	0.44	1.00	0.61
12	24	19	1	4	0.83	0.95	0.88	8	1	15	0.35	0.89	0.50
13	24	14	0	10	0.58	1.00	0.74	12	0	12	0.50	1.00	0.67
14	17	15	0	2	0.88	1.00	0.94	13	0	4	0.76	1.00	0.87
15	28	23	0	5	0.82	1.00	0.90	16	0	12	0.57	1.00	0.73
16	10	8	0	2	0.80	1.00	0.89	9	0	1	0.90	1.00	0.95
17	20	13	0	7	0.65	1.00	0.79	11	0	9	0.55	1.00	0.71
18	31	23	0	8	0.74	1.00	0.85	25	0	6	0.81	1.00	0.89
19	29	27	0	2	0.93	1.00	0.96	22	0	7	0.76	1.00	0.86
20	23	18	0	5	0.78	1.00	0.88	16	0	7	0.70	1.00	0.82
21	21	11	0	10	0.52	1.00	0.69	10	1	10	0.50	0.91	0.65
22	15	12	0	3	0.80	1.00	0.89	11	0	4	0.73	1.00	0.85
23	18	14	1	3	0.82	0.93	0.88	10	1	7	0.59	0.91	0.71
24	18	15	0	3	0.83	1.00	0.91	11	1	6	0.65	0.92	0.76
25	26	17	0	9	0.65	1.00	0.79	13	3	10	0.57	0.81	0.67
26	18	13	0	5	0.72	1.00	0.84	11	3	4	0.73	0.79	0.76
27	29	20	0	9	0.69	1.00	0.82	18	1	10	0.64	0.95	0.77
28	33	22	0	11	0.67	1.00	0.80	17	0	16	0.52	1.00	0.68
29	10	7	1	2	0.78	0.88	0.82	5	1	4	0.56	0.83	0.67
30	27	22	1	4	0.85	0.96	0.90	21	1	5	0.81	0.95	0.88

## Data Availability

Data sharing not applicable.
